# Invisible But Essential: A Qualitative Study on the Experiences of Informal Caregivers in Home-based Palliative Care

**DOI:** 10.1177/00469580251392458

**Published:** 2025-11-14

**Authors:** Samir Husić, Bojan Miletić, Sandra Boskovic, Marica Jerlekovic, Adriano Friganovic, Vedrana Vejzovic

**Affiliations:** 1 Primorsko Goranska County Health Center, Rijeka, Croatia; 2 University of Rijeka, Croatia; 3 University of Applied Health Sciences Zagreb, Croatia; 4 University Hospital Centre Sisters of Mercy, Zagreb, Croatia; 5 University of Malmö, Sweden

**Keywords:** informal caregivers, home-based palliative care, family caregiving, cancer, psychological experience, qualitative

## Abstract

Informal caregivers provide essential support to cancer patients from diagnosis through treatment, palliative care, and end-of-life care. While their practical contributions are widely acknowledged, the emotional and psychological experiences of caregivers remain underexplored, particularly in home-based palliative care settings. Understanding these experiences is crucial to improving support and interventions for caregivers. This study explored the emotional and subjective experiences of informal caregivers providing home-based palliative care for family members with cancer, using retrospective data collected after the patient’s death. A qualitative exploratory study was conducted using semi-structured interviews with 12 informal caregivers recruited from Primorsko-Goranska County, Croatia. Interviews were conducted face-to-face, audio-recorded, transcribed verbatim, and analyzed using thematic analysis to capture patterns and insights in caregivers’ experiences. Analysis revealed one overarching theme, “Still Invisible but Essential,” and 2 major themes: “Confronting the reality of an incurable cancer diagnosis” and “Facing the paradox – choosing between length and quality of life,” encompassing 5 sub-themes. Caregivers described both rewarding and stressful aspects of care, including emotional challenges, communication difficulties, decision-making burdens, and unmet needs for professional support, alongside practical caregiving responsibilities. Informal caregivers are indispensable to home-based palliative care, yet their emotional and subjective experiences are often overlooked. Findings highlight the need for tailored interventions and support services addressing both practical and emotional aspects of caregiving, informing policy, clinical practice, and caregiver support in Croatia and similar contexts.

- Informal caregivers play an indispensable yet often overlooked role in home-based palliative care for cancer patients.
- Caregivers face significant emotional challenges, communication difficulties, and decision-making burdens.
- The study highlights the need for tailored emotional and practical support interventions to improve caregiver well-being and care quality.

## Introduction

Palliative care is a growing global priority, as it seeks to address not only the physical but also the psychological, social, and spiritual needs of patients and their families. The safety, well-being, quality of life, and care of cancer patients inevitably depend on the caregivers who look after them from the time of cancer diagnosis, during active oncologic and palliative treatment, and until the end of life.^1^ They are often referred to as informal caregivers—unpaid individuals, often family members, friends, or others who provide care to someone with a serious illness.^2^ Approximately 10% to 15% of Europe’s population provides informal care, although the definition of caregivers varies across different contexts.^3^ In our context, the commonly used definition is “people without formal healthcare training who care for or support a person with a functional disability, long-term psychiatric or physical illness, or age-related problems.”^4^ Palliative care in Croatia has developed significantly over the past decade, but many challenges remain, particularly regarding home-based care and support for informal caregivers. According to estimates from the Croatian Ministry of Health, approximately 45 000 people in Croatia require palliative care each year, most of whom are cared for at home by family members or close friends. Recent reports indicate that cancer prevalence is particularly high among older adults in Croatia, with malignant diseases being one of the leading causes of morbidity and mortality in this group. Furthermore, national surveys estimate that a substantial proportion of patients with advanced cancer rely on informal caregiving, most often provided by spouses or adult children. Informal caregivers in Croatia, most commonly spouses, adult children, or other relatives, often provide complex care with little formal training or support, and many experience significant emotional, physical, and financial strain. Despite their crucial role in the provision of care, there is limited national data on their experiences, needs, and the challenges they face, particularly in the context of end-of-life care at home. Caregivers’ responsibilities for ill family members increase as cancer progresses, particularly when targeted oncology treatment does not provide the “expected curative outcomes” and when caregivers provide home-based palliative care.^5^ In this context, although the World Health Organization emphasizes the need for a multidisciplinary approach to the patient and highlights the importance of caring for the family and caregivers, most healthcare workers today use the so-called “patient-centered care” approach, which promotes informed and independent decision-making by the patient while neglecting their social environment.^6,7^ It is, therefore, not surprising that the experiences of caregivers are very different. On the one hand, they emphasize positive experiences in caring. On the other hand, they report negative experiences due to pressure from an ill family member, relatives, or health workers to provide home-based palliative care.^8^ Caregivers often overlook the personal consequences of caring for a dying family member, which can lead to fear of death, uncertainty, loneliness, exhaustion, and burnout.^9^

The closeness between the ill family member and the caregiver is crucial for emotional support. When coupled with good communication with professionals and services, it contributes to the relief of pain and other symptoms in palliative care patients.^10^ Caregivers are often an integral part of the patient’s identity and play a key role in providing palliative care to their loved ones.^11^ In their research, Auclair et al emphasize the important role of informal carers, who should be involved in palliative and end-of-life care.^12^ However, they are often unprepared for this role and tend to neglect their own needs, especially when forced to take a more active role in home-based care. Most caregivers have never experienced death and, therefore, need detailed explanations about the dying process.^13^ Sometimes, they feel lost, caught in a dilemma between prioritizing the patient’s needs and neglecting their own life needs or thinking about themselves, which can ultimately harm their quality of life and that of the patient.^14^ Informal caregivers are involved in treatment decisions in different ways. They may play a direct role, by actively contributing to decisions, or an indirect role, by influencing decisions through support or suggestions. Their involvement can be minimal or leading, and their influence can help or hinder the process.^15^ Living with cancer leads to increased emotional and psychological stress for both the patient and the caregivers due to uncertainty about disease progression, recurrence, treatment, and prognosis - mainly due to inadequate psychological support and lack of information about the disease.^16^ The progression of the disease largely determines the intensity of the needs of both the ill family member and their caregivers. In addition to addressing their concerns and uncertainties, the caregivers must also manage the worries throughout the illness, especially during crises, difficult decision-making, and the application of therapeutic procedures accompanied by side effects.^17^ However, the primary task remains assisting the patient with routine daily activities. All these responsibilities can disrupt the caregiver’s mental health and diminish their quality of life.^18^ Professional psychotherapeutic support from a mobile palliative care team (MPT) at home can increase caregivers’ sense of security and stability, alleviate fear, and reduce the anxiety that accompanies them throughout the illness journey.^19^ The MPT in Croatia is a publicly funded service that provides home-based palliative care through a multidisciplinary team, typically composed of a physician, nurse, and work coordinator. Their role is to ensure symptom management, psychosocial support, and coordination of care for patients with life-limiting illnesses and their families. Timely inclusion of professional psychological support, starting at the time of diagnosis and particularly during active oncological treatment and beyond, reduces the risk of developing psychological disorders.^20^

Most patients with incurable malignant diseases share treatment options with their caregivers, making they an essential part of the decision-making process regarding further treatment or the place of end-of-life care.^21,22^ This shared decision-making improves the quality of life for palliative patients and, with the support of an MPT, enhances the quality of palliative care at home.^23^ Healthcare professionals primarily support caregivers by providing education on practical patient care activities, pain and symptom management, access to home medical aids for meeting physical needs, emotional support, and fostering a sense of security through improved accessibility and communication between the caregiver and the MPT.^24,25^ Timely, professional, and effective communication between healthcare professionals, caregivers, and an ill person ensures more efficient management of that person’s physical and psychological difficulties. At the same time, it empowers the caregivers to fulfill their role in home-based palliative care and prepare for end-of-life care at home. This communication begins at the stage of diagnosis, and initial hospital treatment continues through the transition from hospital to outpatient care and extends to palliative care at home, allowing families to prepare for the final stages of life and death.^26^

### Rationale

Although the needs of caregivers are well documented in the literature, their emotional experiences and inner struggles remain insufficiently explored.^27,28^ Despite the central role of informal caregivers, little is known about how they in Croatia experience providing home-based palliative care for a family member. This study therefore explores their subjective experiences, drawing on retrospective accounts collected after the death of their relatives. By examining the caregiving journey in its entirety, the study seeks to provide insights that can guide the development of caregiver support within Croatian palliative care services. Therefore, this study aimed to explore the emotional and subjective experiences of informal caregivers providing home-based palliative care for family members with cancer, using retrospective accounts gathered after the patient’s death.

## Methods

### Study Design

This study is part of a broader mixed-methods research project aiming to explore the experiences, needs, and challenges of informal caregivers providing home-based palliative care in Croatia. The larger project includes both qualitative and quantitative phases and will also examine healthcare professionals’ perspectives and organizational aspects of home-based palliative care delivery. The present study employed a qualitative exploratory approach using semi-structured interviews with caregivers in the home setting. Given the limited research on informal caregivers’ experience in the Croatian context regarding home-based palliative care, a qualitative exploratory design was chosen to gain an in-depth understanding of caregivers’ lived experiences, which could not be captured through structured questionnaires.

Qualitative thematic analysis was used during data analysis.^29^ This method was chosen because it offers a flexible and systematic approach to identifying, analyzing, and reporting patterns (themes) within qualitative data. The study followed the relevant EQUATOR guidelines for qualitative research, and the research protocol was developed following the Consolidated Criteria for Reporting Qualitative Research (COREQ) guidelines for qualitative studies.^30^

### Setting

The present study was conducted in Primorsko-Goranska County, Croatia (approx. 108 000 inhabitants, according to the 2021 census), where patients can choose home-based palliative care supported by a multidisciplinary team (MPT). In Croatia, the MPT is a publicly funded service that provides home palliative care through a team usually consisting of a doctor, a nurse, and a care coordinator. Their role is to provide symptom management and psychosocial and spiritual support. The MPT coordinates the care of persons with life-limiting illnesses and their families.

### Participants

Twelve informal caregivers were recruited after the death of their relatives, who had received home-based palliative care. The interviews were conducted between December 2023 and May 2024. Recruitment began after ethical approval was obtained from the Primorje-Gorski Kotar County Health Center (No. 01-286/1-2-23, May 9, 2023). Participants were identified and approached by healthcare professionals (nurses and physicians) from the multidisciplinary palliative team (MPT) who had previously provided care to the deceased persons. The responsible nurse contacted the bereaved caregivers by phone approximately 6 to 12 weeks after the person’s death, following an assessment of appropriateness given the grieving processin. A total of 12 caregivers were invited, and all agreed to participate. The study included adult caregivers (≥18 years) in a home-based palliative care setting who met the definition of caregiver for this study, which means: provided care at home for a relative receiving palliative care, the cared-for person had died within the past 6 to 12 months, ability to provide informed consent and participate in an interview in Croatian.

Exclusion criteria were: caregivers with cognitive impairments that would prevent meaningful participation. The small sample size (n = 12) was deemed sufficient for in-depth narrative analysis, and data saturation was achieved, as the final 2 interviews did not provide new information.

Basic demographic data on the caregivers and the persons they cared for are presented in Table 1.

**Table 1.** Basic Data of Caregivers and the Person They Cared for.

Caregivers	
Men—N (%)	4 (33.3%)
Women—N (%)	8 (66.7%)
Age (years) M ± SD	54.8 ± 12.92
*Kinship*	
Spouse—N (%)	9 (75%)
Daughter—N (%)	3 (25%)
Time from patient death to interview with IC (days) M ± SD	71.58 ± 24.60
Family member	
*Gender*	
Men—N (%)	6 (50%)
Women—N (%)	6 (50%)
Age (years) M ± SD	60.6 ± 10.98
Time from diagnosis to first visit of the MPT (months) M ± SD	45.8 ± 42.9
*Place of death*	
Home—N (%)	9 (75%)
Institution—N (%)	3 (25%)

*Note.* N = number; % = percentage; M = mean; SD = standard deviation.

### Data Collection

Semi-structured interviews were conducted face-to-face in the caregiver’s home by a healthcare professional experienced in palliative care (SH). Guidance was provided by an experienced qualitative researcher (VV) to ensure consistency and methodological rigor. The first interview was a pilot interview to refine the interview guide and techniques, conducted by the first author in consultation with the last author. After review and discussion, minor adjustments to questions and prompts were made to enhance clarity and data richness.

Before the study, all potential participants were contacted by a nurse from the MPT. They received written and verbal information about the study, time for reflection, and the opportunity to ask questions. Written informed consent was obtained before scheduling interviews at a convenient time and place.

Interviews were audio-recorded using a telephone disconnected from the internet. Interviews began with demographic questions (gender, age, education), and such as “Can you explain that?” and “Can you tell me a little more?” were used to elicit rich narratives and minimize recall bias. The first introductory question was: “Can you tell us about your experience of providing palliative care to your sick relative at home?” Interviews ranged from 16 to 46 min (average 25 min). The interview guide is provided in Appendix 1.

### Data Analysis

All interviews were transcribed verbatim and anonymized. Transcripts were stored securely on a password-protected server.

The first and last authors read all transcripts and listened to recordings to familiarize themselves with the data. Open coding was conducted independently by the first 2 authors. Codes were compared and iteratively grouped into themes through team discussions to ensure internal coherence and external heterogeneity. The final themes were reviewed and agreed upon by the full author group, which included members with diverse expertise to reduce bias and ensure multiple perspectives. Table 2 presents the process of defining themes and example quotes (Appendix 2).

## Results

A total of 12 caregivers were interviewed. The average age was 54.8 years and ranged from 30 to 74 years. Most of the caregivers were women (8 or 66.7%). Spouses accounted for 9 (75%) of the caregivers. The time between the patient’s death and the interview averaged 71.58 days, with a minimum of 36 and a maximum of 99 days. The average age of the patient was 60.6 years, with a minimum of 40 and a maximum of 80 years. The time of contact with palliative care after diagnosis averaged 45.8 months, with a minimum of 3 and a maximum of 132 months. The deceased care recipient had been diagnosed with a variety of advanced-stage cancers, including lung cancer (n = 6), colorectal cancer (n = 1), pancreatic cancer (n = 1), breast cancer (n = 3), and malignant melanoma (n = 1).

Results of the study are summarized in one overarching theme, “Still Invisible but Essential,” and 2 major themes: “Confronting the reality of an incurable cancer diagnosis” and “Facing the paradox – choosing between length and quality of life,” comprising 5 sub-themes.

### Overarching Theme: Still Invisible But Essential

This theme captures caregivers’ experiences of feeling overlooked and undervalued despite playing a crucial role in supporting their loved one. Their contribution, though indispensable, often lacks formal recognition, resources, and inclusion in care planning. Caregivers described a symbiotic relationship with the patient, forming a dyadic bond shaping the caregiving experience.

Caring for a person with incurable cancer at home was emotionally and physically demanding, particularly for those without prior caregiving experience or knowledge of symptom management, emotional support, and end-of-life care.

The role extended beyond practical tasks to managing their own emotions while addressing the person’s needs, often amid differing expectations and wishes. As the illness progressed, caregivers faced increasingly complex decisions, some conflicting with personal beliefs or the person’s earlier preferences. The relationship dynamic shifted from equal partnership to a more dominant caregiving role, with responsibility for decision-making. While necessary to ensure appropriate care, this shift often brought emotional distress, as caregivers struggled to balance respect for autonomy with the need to act in the person’s best interests.

## Theme: Confronting the Reality of an Incurable Cancer Diagnosis

This theme describes the profound emotional impact of hearing that a loved one’s illness is incurable. It encompasses the shock, grief, and sense of helplessness that arise at the moment of diagnosis, as well as the initial struggle to process and accept this new reality.

### Sub-Theme: The Struggle Between Fear and Hope

Caregivers experienced an ongoing tension between fear and hope, affecting communication with the patient. The sudden intrusion of the unknown and the loss of control often impeded rational reflection. The inner struggle between fear and hope was a pervasive experience among caregivers and simultaneously posed significant challenges in their communication with the person they cared for.

The sudden intrusion of the unknown and the feeling of losing control created a paralyzing effect for both parties, often impeding rational reflection on the situation. From the moment the disease was diagnosed, the word “cancer” dominated thoughts and conversations, evoking deep fear and uncertainty.

Despite these fears, caregivers found themselves caught in a dilemma—balancing their apprehensions with the need to provide support. One caregiver recalled receiving distressing news: This revelation left her in shock and temporarily incapacitated her ability to manage daily life, even as she was expected to support the person in greatest need:

(IC 06, wife) “I was shocked, I just started crying and was completely overwhelmed. I was supposed to go to work that afternoon, but I wasn’t able to do anything. . . I couldn’t even think.”

At the same time, caregivers sought to overcome fear through a shared hope for a positive outcome and recovery. This hope, while often unrealistic in the face of late diagnosis, metastases, and limited treatment options, served as an important coping mechanism:

(IC 02, husband) “She accepted the diagnosis with the hope that she would make it. Her hope was much higher than it would have been if she had known the real situation. . . she was not fully aware of the severity of the disease.”

Although some informal caregivers were more realistic about the prognosis, they concealed their doubts to maintain hope for a miracle. Caregivers also described how some family members coped with fear through emotional withdrawal and silence. This “personal silence” often masked thoughts about limited remaining time, sometimes compounded by ambiguous or pessimistic prognoses communicated by healthcare professionals:

(IC 01, wife) “He was silent, kept everything to himself, didn’t talk much about it. He didn’t even talk to me. He asked the oncologist, 'Doctor, how much time do I have left?’ And the doctor told him, 'Three to four weeks.’ He wasn’t angry or nervous—he was just a person who kept everything to himself, quietly.”

Despite these challenges, caregivers expressed a deep commitment to their loved ones, an enduring sense of shared destiny, love, and devotion, and a strong desire to support them until the end. Some caregivers openly shared their fears and insecurities with the person they cared for, sometimes crying together:

(IC 10) “There was this overwhelming insecurity, something unknown. She was sitting there, and I went to her, squatted down, and hugged her. . . she was crying, very quietly.”

Conversely, others refused to accept the diagnosis and fought resolutely, often directing their anger toward the healthcare system, which they felt had failed to act promptly or effectively:

(IC 05) “How is this possible? Death—unbelievable. . . but we will not give up. . . we will fight. . . we will not accept what they say.”

Throughout these struggles, caregivers were expected to provide continuous support to the person they cared for, despite often unclear guidance on how best to do so.

### Sub-Theme: Communication Challenges and Their Impact on Caregivers

Caregivers reported that technical communication of diagnosis often lacked emotional attunement, increasing distress. Abrupt delivery, medical jargon, or restricted access led to confusion, frustration, and feelings of isolation.

The communication of a cancer diagnosis emerges as a pivotal moment shaping the trajectory of caregivers’ engagement with healthcare professionals and their emotional coping. Our findings indicate that while the transmission of clinical information typically occurred in hospital settings by oncologists or emergency physicians, this “technical” delivery often lacked the emotional attunement necessary to support caregivers facing life-altering news.

Several caregivers reported feeling overwhelmed or cognitively incapacitated upon receiving the diagnosis, even when healthcare providers attempted compassionate communication. This highlights a tension between the clinical imperative to convey clear information and the psychological readiness of recipients to process such data:

(IC 05) “The doctor explained the surgery, but I was in shock and couldn’t absorb the details.”

The data reveal that inadequate communication skills—manifested as abruptness, use of medical jargon, or insufficient empathy, were a primary source of dissatisfaction and emotional distress among caregivers. Caregivers expressed a need for information that was not only accurate but also conveyed with sensitivity and reassurance, underscoring the critical role of communication style in mediating caregivers’ psychological adjustment:

(IC 03) “The oncologist’s tone was professional yet harsh, lacking human warmth.”

(IC 02) “The doctor’s quick mention of ‘suspected cancer’ felt like a casual dismissal of a devastating reality.”

Moreover, the exclusion of caregivers from direct communication due to institutional restrictions, such as COVID-19 protocols, exacerbated feelings of isolation and helplessness:

(IC 05) “Being outside when the diagnosis was delivered felt like a cruel barrier.”

Conversely, when communication was direct, succinct, and perceived as appropriately timed, caregivers reported greater satisfaction, highlighting the importance of clinicians’ assessment of the recipient’s emotional state and readiness:

(IC 07) “The oncologist’s straightforward approach felt honest and respectful.”

However, even well-intended communication faltered when overwhelmed by medical complexity, leading to confusion and incomplete understanding:

(IC 10) “Conflicting statistical information left us uncertain about prognosis.”

Throughout the treatment journey, caregivers struggled to maintain consistent contact with medical teams, with sporadic availability of healthcare professionals contributing to feelings of frustration and abandonment:

(IC 12) “We were left without explanations for long periods, deepening our anxiety.”

The lack of empathic engagement during critical moments, such as disease progression, further compounded caregivers’ distress and sense of disenfranchisement:

(IC 06) “Denied timely consultation, I felt powerless and devastated.”

Some caregivers attempted to mitigate these gaps through informal networks, illustrating the reliance on social capital to navigate healthcare complexities:

(IC 12) “A family friend who was a doctor helped clarify information for us.”

Finally, the practice of delivering diagnoses primarily to caregivers rather than caring person, often with subsequent filtering and paternalistic control over information sharing, raises ethical considerations regarding autonomy and informed decision-making, potentially influencing treatment perceptions and choices.

These findings underscore that effective communication in oncology care must transcend mere transmission of facts, encompassing emotional support, contextual sensitivity, and ongoing engagement to empower caregivers and patients alike.

## Theme 2: Facing the Paradox—The Choice Between Life’s Length and Quality

Caregivers were often caught in paradoxical situations, as the person receiving care shifted between wanting to prolong life and prioritizing its quality. These changes were linked to the person they cared for, physical state—severe symptoms often brought a more negative outlook, while relief renewed the desire to keep fighting. Caregivers tried to adapt but found the constant emotional shifts exhausting. Many felt isolated in their efforts to meet the changing wishes of the person they cared for. This ongoing struggle weighed heavily on their emotional and physical well-being.

### Sub-Theme: The Burden of Decision-Making

Treatment decisions—particularly regarding whether to continue or stop aggressive therapy- emerged as a central source of emotional stress for caregivers. Caregivers often felt caught between respecting the patient’s wishes, understanding medical recommendations, and managing their own hope and fear. The emotional toll was amplified when patients insisted on continuing treatment despite severe side effects, as illustrated by one caregiver:

“We carried on to the end. Then came the results—metastases all over his liver. Then he gave up.” (IC 06)

In these situations, caregivers experienced a complex mixture of hope, guilt, and anticipatory grief, often feeling responsible for supporting decisions that could significantly affect the patient’s quality of life. Conversely, when patients refused treatment outright, caregivers sometimes felt relief but also uncertainty, struggling with doubts about whether the patient had fully understood the implications:

(IC 08) “She said, ‘I don’t want chemotherapy.’ The doctor documented it, and that was it.”

Some patients accepted the absence of further treatment calmly, which provided caregivers with a sense of reassurance and emotional stability:

(IC 02) “She bravely accepted it—no panic, no reaction.”

However, avoidance of discussions about prognosis or treatment often concealed deep emotions. Caregivers reported that patients’ unspoken fears and unexpressed desires could surface unexpectedly, increasing caregiver anxiety and emotional burden:

(IC 11) “She once told me, crying, ‘I still want to live. . . for you.’”

These narratives highlight that decision-making in palliative care is rarely straightforward. Caregivers are not only witnesses to the patient’s choices but also active participants in navigating hope, fear, and the practical implications of care. The burden is compounded when caregivers feel insufficiently informed or unsupported by the healthcare team, emphasizing the need for structured communication, timely information, and emotional support to help caregivers manage both the practical and emotional challenges of end-of-life decision-making.

### Sub-Theme: Psychological Professional Support—Yes or No

Caregivers experienced significant psychological stress throughout the illness, but opinions on professional support varied. Stress often started early, intensified during crises, and peaked near death. Few caregivers accessed professional psychological help, finding it valuable, while most either declined it or were unaware it existed:

(IC 07) “We didn’t get any support, we didn’t even know it existed. . . And I think we both would have needed it.”

Many relied on family or friends, or patients coped through work or activities:

(IC 09) “He found strength in his work and used it as an outlet. . . ‘I’m saving money because when that day comes, you have nothing to go on with.’ That was his way until the end.”

Some caregivers focused solely on the patient’s physical needs, delaying emotional support for themselves:

(IC 02) “At that moment, the most important thing for me was to be with my son. And we had to help her. . . She couldn’t do anything on her own.”

After the patient’s death, some became receptive to professional support and found it beneficial:

(IC 05) “After she died, we got the phone number of a psychologist. And she helped us a lot. . . I couldn’t stop crying for an hour.”

Some caregivers felt psychological support was unnecessary for themselves due to personality or coping style:

(IC 06) “They felt such sessions were unnecessary for themselves as they were ‘not that type’. . . which led them to reject the psychological support offered at home.”

### Sub-Theme: Support From the Multidisciplinary Palliative Team (MPT)

Caregivers’ trust in the MPT developed over time, enhancing confidence and satisfaction. Initial doubts or lack of awareness of services were common. During active treatment, family physicians and MPTs were often involved only in administrative tasks. Many caregivers were unaware that MPTs could visit at home to support both patient and caregiver:

(IC 02) “I didn’t even know you (MPT) existed. . . I had no choice but to go to the doctor and ask for a referral.”

Caregivers constantly balanced prolonging life with preserving quality, navigating ethical, emotional, and practical complexities that carried profound consequences for both patient and caregiver.

Family members not involved in daily care sometimes resisted accepting the ill person’s decline:

(IC 05) “My older son. . . didn’t understand. . . he said: ‘Take her somewhere, get them treated, do something.’”

Patients often delayed accepting MPT services until symptoms became severe:

(IC 04) “The medication was no longer working. . . Finally, he told me, ‘Let’s try, call them (MPT).’ That was the turning point.”

When trust developed, caregivers felt more confident seeking help:

(IC 01) “I felt more confident because I knew I could contact you (MPT) by phone. . . I could share my difficulties and get guidance.”

Initially, some doubted home-based care without hospital equipment, but clear communication and symptom relief built trust and satisfaction:

(IC 04) “He had been bedridden. . . We wanted to do it together. And we did—to alleviate his suffering.”

Caregivers constantly navigated the tension between prolonging life and preserving its quality, balancing patient wishes, family dynamics, and available support. This often left them carrying the weight of decisions with profound consequences for both the patient and themselves.

## Discussion

Analysis of this study, which aimed to gain insight into the experiences of caregivers providing home-based palliative care, revealed dissatisfaction among caregivers with their role in caring for a person they care for. Caregivers expressed feelings of loneliness, struggled between fear and hope, and often experienced anger and helplessness. These experiences were captured in the overarching theme *“Still Invisible but Essential,”* reflecting how caregivers perceived themselves as indispensable in practice but overlooked by the healthcare system. Such feelings were closely related to a lack of information about the person they cared for’s condition and the slow response of the healthcare system to their needs. Despite the development of home-based palliative care, a lack of understanding of caregivers’ needs persists, a concern also highlighted by previous studies.^14,15^

Our findings illustrate that these challenges were intensified by the paradox caregivers faced in “choosing between length and quality of life,” where they struggled to support the person’s wishes while coping with treatment side effects and declining health. Unrealistic expectations regarding treatment options further increased stress and exhaustion, which likely impacted the quality of care they provided. Early involvement of a multidisciplinary team (MPT) in home care has been shown to support both caregivers and patients,^31^ and a higher level of professional support could help caregivers feel less isolated. Late involvement and incomplete information have been associated with feelings of uselessness, helplessness, inadequacy, and lack of control.^32,33^

### The Role of Caregivers and Need for Reassurance

Caregivers often need reassurance that they are performing caregiving tasks correctly, a finding consistent with previous research.^34^ Our study adds nuance to this by showing that such needs are not only practical but also deeply emotional, as caregivers simultaneously managed their own fear, hope, and grief. However, their role cannot be seen in isolation from the wider social context in which caregiving is not officially recognized.^35^ Caregivers demonstrate a desire to fulfill the patient’s wishes regarding medical treatments, often facing dilemmas about the extent to which these wishes should be followed. Improved communication between caregivers and medical staff remains a critical need.^36^

Caregivers are never fully prepared for the impending death of the person they care for, regardless of the duration of caregiving or location of death.^37^ Conversations aimed at preparing caregivers for the patient’s death, although rare, could reduce stress, alleviate anger and sadness, and help navigate grief. Our findings support that early and regular involvement of MPT, rather than only during the terminal phase, could facilitate better preparation for treatment decisions and anticipated loss.

## Hope, Treatment Decisions, and Communication

Caregivers often attempt to maintain hope for recovery, even when it is not realistic due to delayed consultations, metastatic diagnoses, or limited treatment options. Similar findings were reported in Croatia, where caregivers expressed sadness, anger, and anxiety but directed their energy toward hope.^38^ In the present study, this hope functioned as both a coping strategy and a source of conflict, as it was frequently at odds with medical recommendations. Uncertainty regarding treatment continuation, especially when discontinuation is suggested, increased caregivers’ dissatisfaction, as it was seen as a loss of hope.^39^

The delivery of bad news is delicate, and caregivers prefer clear, compassionate communication, with reassurance that “all will be well.” Cultural and linguistic appropriateness is critical.^40,41^ Some caregivers were unhappy with the directness of communication, while others appreciated clear and professional delivery, reflecting the patient’s character and preferences. A lack of empathy or time from medical staff adds to caregivers’ suffering, as they seek hope and emotional support. These findings echo our sub-theme on “communication challenges and their impact,” showing that the style of communication shaped caregivers’ trust and long-term coping Caregivers may avoid discussing prognosis to protect themselves emotionally, while others insist on speaking to doctors to influence decisions on the patient’s behalf.^42^

## Psychological Support and Coping Strategies

Lack of timely information and psychological support, compounded by focus on treatment and prolonging the patient’s life, often leads caregivers to neglect their own psychological needs. Support is often realized as necessary only after the patient’s death. Timely psychological support could contribute to caregiver stability, and in some cases, may be more important than information itself.^43^ Our study highlights that caregivers’ acceptance or rejection of professional support depended strongly on personality, coping style, and the timing of the offer.

Focusing on activities outside caregiving, such as work or exercise, helps caregivers maintain a positive attitude and gain psychological relief .^44^ Yet these strategies also reveal dual roles, as family members and as primary cares, which created fluctuations between closeness, anxiety, and emotional distance.^45,46^ Access to counseling, group support, and community resources is crucial for improving conditions in home care.^47^

## Access and Utilization of Support Services

Our results show that although hospitals have specialists trained in psychological care, these services are rarely used during oncological treatment. In-home care access was sporadic, often dependent on caregivers’ awareness rather than systematic inclusion in treatment. This reflects their perception of being “invisible,” despite their essential role. Pilot studies, including psychoeducational interventions, demonstrated improved preparation, awareness of needs, and confidence in interacting with the patient.^48^ Croatian studies similarly show that caregivers require support to maintain their health and social inclusion.^49^ Applying learned skills in daily life may be challenged by cultural, sociological, and individual factors.

## Strengths and Limitations of the Study

This study has both strengths and limitations. A strength is that it was conducted with caregivers providing home-based palliative care, for a person with incurable cancer, regardless of the type of cancer. This allowed for variation in experiences. On the other hand, a limitation may be that the study was conducted after the person’s death, and this loss may have influenced the caregivers’ experiences. However, this can also be seen as a strength, as the caregivers were no longer exposed to the daily stress of caring for the ill person.

Both spouses and children of the person they cared for participated in the study, which could be seen as a limitation, as their connection to the person was not on the same level. However, it can also be regarded as an advantage, as it provides a broader view of the experiences. The study did not examine the impact of parameters such as the caregiver’s relationship with the person, gender, education, or employment status, and no comparison was made based on these criteria. Because only caregivers were interviewed, the perspectives of both the caregiver and the person they cared for are filtered through the caregiver’s viewpoint. The study was conducted exclusively with caregivers of persons who died of cancer and not of other non-cancer-related illnesses, which may limit the overall understanding of the topic.

## Clinical Implications

Caregivers of patients with terminal cancer require early, structured support from multidisciplinary teams to reduce emotional burden and improve care quality. Clear, compassionate communication about the patient’s condition and treatment options is essential to reduce uncertainty and support shared decision-making. Systematic access to psychological counseling and practical guidance can enhance caregivers’ confidence and coping skills. Recognizing caregivers as key partners in palliative care and tailoring support to their cultural and individual contexts are crucial. Integrating these strategies into home-based palliative care can improve both caregiver well-being and patient outcomes.

## Conclusion

Informal caregivers play a multifaceted role when providing palliative care at home, facing not only the physical demands of caregiving but also navigating complex emotional and psychological challenges. Beyond assisting with daily activities and symptom management, they are deeply involved in decision-making processes, offering continuous emotional support, and coping with the uncertainty surrounding the progression of the illness. The weight of these responsibilities can be overwhelming, particularly as the caregiver must balance their own emotions with the needs and expectations of the persons they care for. Given these challenges, targeted interventions are crucial to support caregiver in their role. Timely access to professional psychological support, structured educational programs, and effective communication between healthcare providers and caregivers can help alleviate their burden. Implementing such interventions can improve caregivers’ well-being, enhance the overall caregiving experience, and contribute to better palliative care outcomes.

## Supplemental Material

sj-pdf-1-inq-10.1177_00469580251392458 – Supplemental material for Invisible But Essential: A Qualitative Study on the Experiences of Informal Caregivers in Home-based Palliative Care Supplemental material, sj-pdf-1-inq-10.1177_00469580251392458 for Invisible But Essential: A Qualitative Study on the Experiences of Informal Caregivers in Home-based Palliative Care by Samir Husić, Bojan Miletić, Sandra Boskovic, Marica Jerlekovic, Adriano Friganovic and Vedrana Vejzovic in INQUIRY: The Journal of Health Care Organization, Provision, and Financing

## Appendix 1: Interview Guide

### The Questions for the Caregivers

Can you describe your role in providing care for your family member with cancer?

How long have you been caring for them, and how many hours per day do you dedicate to caregiving?

What types of support do you provide (physical, emotional, administrative, etc.)?

### Emotional and Psychological Aspects

How would you describe your emotional state since taking on the caregiving role?

What are the biggest emotional challenges you face in this role?

How do you cope with stress and emotional pressure related to caregiving?

### Challenges and Barriers in Caregiving

What are the biggest challenges you encounter in providing care?

Have you had access to professional support (healthcare professionals, counseling, support groups)? If so, how helpful has it been?

Are there situations where you feel overwhelmed or unsure about how to provide care?

### Social and Economic Impact

How has caregiving affected your daily life and your relationships with other family members?

Has caregiving had an impact on your financial situation? If so, in what way?

How do people around you (friends, extended family, community) react to your role as a caregiver?

### Needed Support and Improvements

What kind of support would you like to have to make your caregiving role easier?

What do you think the healthcare system could improve to better support informal caregivers like you?

If you could change something about the support you receive, what would it be?

### Final Reflections

What have you learned from this experience?

What would you say to other caregivers going through a similar situation?

Is there anything else you would like to share that you feel is important?

## Appendix 2

**Table 2.** The Process of Defining Themes and Subthemes with Example Responses.

Theme	Subtheme	Code	Quotations
THEME 1. Confronting the reality of an incurable cancer diagnosis	The struggle between fear and hope	Fear of death	*(IC 05) “How is this possible? Death — unbelievable, but we will not give up. . . we will fight. . . we will not accept what they say. It’s shocking. She knew beforehand that something was wrong. They just told us it was nothing. . . that it was ‘misread’ (sarcasm).”* *(IC 06) “I won’t be coming home anytime soon. I have to have an operation. Cancer — I’ve always been afraid of that word. I never thought about this disease like this. . .that it could be so aggressive.”*
		Hope he gets out.	*(IC 02) “She accepted the diagnosis with the hope that she would make it. Her hope was much higher than it would have been if she had known the real situation. . .she was not fully aware of the severity of the disease.”* *(IC 01): “I was always there for him, always supported him, and gave him hope that he would make it.”*
	Communication challenges and their impact on caregivers	Clear, correct, professional – good conversation	07*(IC 07) “The oncologist told us, correctly and professionally. We were satisfied with the way it was communicated to us. It was a short conversation. The most important thing is that the doctor felt it was the right way to tell him, directly, to the point /. . ./”*
		Kindly but unclear	*(IC 10) “The oncologist said something, but we didn’t quite understand it. He was kind and compassionate /. . ./. Then the surgeon mentioned something about a 50 percent chance of recurrence after 50 years, but I didn’t understand that.”*
		Professional, rough, raw	*(IC 03) “The oncologist told us in the office, very professionally, like she was just telling us the time. . .It was clear, but somehow inhuman, harsh, raw.”*
THEME 2. Facing the Paradox – The Choice Between Life’s Length and Quality	The burden of decision-making	We didn’t talk about it.	*(IC 02): “I think she accepted it bravely. . .. When the radiotherapy was over and she was told that there were too many metastases, she just bravely accepted it. We’ll hang in there until the end, however long it takes. No panic, no reaction.”* *(IC 11): “We didn’t talk about it much, at least not to me. It was like she had been written off, and I think that was hard for them too. . .I just remember her telling me once (pause, crying), ‘I still want to live. . .. for you.”*
		He said he had to go all the way and we did.	*(IC 06): “They started chemotherapy. At* first, *the first two treatments were fine but then came the vomiting and everything else. But we carried on to the end. He said he had to keep going, he had to hang in there. Then we got the results. . .Metastases all over his liver. Then he collapsed and gave up.”* *(IC 07) “He had hoped for therapy. . .. The doctor told him that further treatment was not necessary. He insisted, but he was very weak. They said he could come for a checkup, so I had to go with him.”*
		She seemed relieved She said she didn’t want it anymore	*(IC 08): “When we had the consultation, she said, ‘I don’t want chemotherapy.’ The doctor said, ‘We have to offer you the treatment, but you’ve made your decision and I have to document it.’ That was fine, but then she added: ‘Sometimes patients are smarter than doctors.’”*
	Psychological professional support yes or no	We didn’t know about it.. Only after death..	*(IC07): “We didn’t get any support, we didn’t even know it existed, although I think it would have been good. /. . ./ We got nothing, absolutely nothing /. . ./ And I think we both would have needed it.”* *(IC05): “You know, after she died, we got the phone number of /. . ./, a psychologist. And she helped us a lot. /. . ./ I couldn’t stop crying for an hour. Oh, how much A. (FM) would have benefited if she had had therapy.”*
		Second focus: work and family	*(IC02) “I was with my younger son who lives here (in the apartment with us). I just needed us to support each other. I didn’t feel the need to talk to a professional. At that moment, the most important thing for me was to be with my son. And we had to help her (FM). She couldn’t do anything on her own, not even eat.”* *(IC09):“/. . ./ He found strength in his work and used it as an outlet. He took his tools and his car and went to work. I kept telling him not to do it, but he said, /. . ./ ‘I’m saving money because when that day (death) comes, you have nothing to go on with.’/. . ./ That was his way until the end.”*
		It helped me a lot; he refused	*(IC10): “There was chaos, complete disorder (after the patient’s death). I attended individual and group therapy sessions for several months before she passed away. /. . ./ I went every week. I had a group /. . ./ I had psychological support /. . ./ Group support. So I felt very supported in that way. And that gave me a rhythm, it brought me back /. . ./ It was support.”* *(IC06): “They felt such sessions were unnecessary for themselves as they were “not that type” due to their personality (reserved nature), which led them to reject the psychological support offered at home.“*
	Support from MPT at home	We didn’t know you existed.	*(IC02) described the moment he decided to contact the MPT*: *"I didn’t even know you (MPT) existed. When my wife couldn’t eat or drink for days, I had no choice but to go to the doctor and ask for a referral to the hospital or ask if someone could come /. . ./.*
		We only called when there was no help in sight.	*(IC04): “The reason (for calling the MPT) was that the medication was no longer working. The pain wouldn’t stop, nothing helped. He kept putting it off, but finally, he told me, ‘Let’s try, call them (MPT).’/. . ./ That was the turning point.”* *(IC05) explained: "My older son was here, the younger one was abroad. He didn’t understand /. . ./ I didn’t know how to explain it to him without scaring him. He said: ‘Take her somewhere (to hospital), get them treated, do something“*
		We could always rely on you.	*(IC01): “I needed support, help /. . ./ I felt more confident because I knew I could contact you (MPT) by phone. I trusted you that I could share my difficulties with someone /. . ./ that you could give me advice and I could help him through your guidance.”* *(IC03):“". . .Every time I called, you were there. . .each time I got an explanation, instructions, understanding.”*
